# Multiparametric radiomics methods for breast cancer tissue characterization using radiological imaging

**DOI:** 10.1007/s10549-020-05533-5

**Published:** 2020-02-04

**Authors:** Vishwa S. Parekh, Michael A. Jacobs

**Affiliations:** 1 The Russell H. Morgan Department of Radiology and Radiological Science, The Johns Hopkins School of Medicine, Baltimore, MD 21205 USA; 2 Sidney Kimmel Comprehensive Cancer Center, The Johns Hopkins School of Medicine, Baltimore, MD 21205 USA; 3 Department of Computer Science, The Johns Hopkins University, Baltimore, MD 21208 USA

**Keywords:** Breast cancer, Radiomics, Texture, Informatics, Machine learning, Magnetic resonance imaging, Multiparametric imaging, Diffusion, ADC, Entropy, Gray-level co-occurrence matrix (GLCM)

## Abstract

**Background and purpose:**

Multiparametric radiological imaging is vital for detection, characterization, and diagnosis of many different diseases. Radiomics provide quantitative metrics from radiological imaging that may infer potential biological meaning of the underlying tissue. However, current methods are limited to regions of interest extracted from a single imaging parameter or modality, which limits the amount of information available within the data. This limitation can directly affect the integration and applicable scope of radiomics into different clinical settings, since single image radiomics are not capable of capturing the true underlying tissue characteristics in the multiparametric radiological imaging space. To that end, we developed a multiparametric imaging radiomic (mpRad) framework for extraction of first and second order radiomic features from multiparametric radiological datasets.

**Methods:**

We developed five different radiomic techniques that extract different aspects of the inter-voxel and inter-parametric relationships within the high-dimensional multiparametric magnetic resonance imaging breast datasets. Our patient cohort consisted of 138 breast patients, where, 97 patients had malignant lesions and 41 patients had benign lesions. Sensitivity, specificity, receiver operating characteristic (ROC) and areas under the curve (AUC) analysis were performed to assess diagnostic performance of the mpRad parameters. Statistical significance was set at *p* < 0.05.

**Results:**

The mpRad features successfully classified malignant from benign breast lesions with excellent sensitivity and specificity of 82.5% and 80.5%, respectively, with Area Under the receiver operating characteristic Curve (AUC) of 0.87 (0.81–0.93). mpRad provided a 9–28% increase in AUC metrics over single radiomic parameters.

**Conclusions:**

We have introduced the mpRad framework that extends radiomic analysis from single images to multiparametric datasets for better characterization of the underlying tissue biology.

**Electronic supplementary material:**

The online version of this article (10.1007/s10549-020-05533-5) contains supplementary material, which is available to authorized users.

## Background

Radiomics use texture features to define potential quantitative metrics from radiological images [1–11].The texture features extracted are based on several properties inherent to image data, such as, gray-level distribution [12], inter-voxel relationships [13–17], and shape [18]. The goal of radiomics is to provide a quantitative framework for a radiological biopsy of tissue, which could be correlated to the underlying tissue biology. Reviews of several studies that have employed radiomic analysis have produced encouraging results for characterization of different imaging modalities and pathologies in brain, breast, lung, and prostate [9, 19, 20].

However, current radiomic methods are based on extraction of textural features from a single imaging parameter, such as, computed tomography (CT), T_1_- or T_2_-weighted magnetic resonance imaging (MRI), or positron emission tomography (PET) and do not extract the textural features from multimodal or multiparametric radiological datasets consisting of multiple imaging sequences, for example, multiparametric MRI (mpMRI) such as, proton density (PD), T_2_-weighted(T_2_), T_1_-weighted(T_1_), diffusion-weighted (DWI) with apparent diffusion coefficient (ADC) of water mapping, and dynamic contrast enhanced (DCE). These MRI sequences produce different soft tissue contrast of the tissue, where each imaging sequence provides a specific representation of the tissue based on the physics and gray levels in the image.

Prior work in radiomics has primarily focused on extracting radiomic values or features from individual parameters and combining them for “multiparametric” (mp) characterization of selected tissue types into a final model using advanced machine learning or dimensionality reduction techniques [8, 21–26]. Tiwari et al. used machine learning methods with a Support Vector Machine (SVM) in a set of 58 patients (43 for training and 15 validation) with both primary and metastatic brain lesions imaged with brain mpMRI consisting of anatomical imaging parameters of *T*_1_, *T*_2_, and Fluid-attenuated inversion recovery (FLAIR). Regions of interest (ROI) were drawn on lesions from the single parameter images to derive the radiomic features. The Area Under the ROC Curve (AUC) were evaluated, but the AUCs were very low for each of the anatomic radiomic MRI parameters ranging from 0.54 to 0.79 (*T*_1_-Post-Contrast and FLAIR, respectfully) [21]. Li et al. had similar results in a larger patient cohort of 193 cases using radiomic features derived from anatomic imaging parameters of *T*_1_, *T*_2_, FLAIR, and *T*_1_-Post-Contrast [25]. All radiomic features were non-diagnostic with AUCs ranging from 0.61 to 0.71. However, when applying machine learning methods coupled with a random forest classifier to the radiomic feature set, the AUC improved to 0.88 [25]. Similarly, Liu et al. using single radiomic features from *T*_2_, DCE, and DWI in a large dataset of 584 patients with locally advanced breast cancer (LABC) used radiomics to predict treatment response from a combination of single radiomic features and a SVM method [26]. The application of the SVM resulted in a higher AUC of 0.79 by combining each radiomic signature from the mpMRI. However, they used a radiomics of multi-parametric magnetic resonance imaging model that combined single radiomic features and reported an increase in the AUC to 0.86 [26]. The main drawback of these techniques is that they merely combine the textural information from single radiomic features and do not capture the true texture of the underlying tissue characteristics simultaneously. Multiparametric imaging methods are used to interrogate different soft tissue contrasts of the tissue for improved characterization of each tissue type. These different tissue contrasts provide a specific representation of each tissue type-based physiological properties and physics within the tissue and the image. The integration of all imaging information from these different radiological parameters provides a more complete view of the underlying biological tissue characteristics. Correspondingly, texture analysis on the complete multiparametric datasets would provide information about the “true texture” of the tissue rather than from a single specific point of view or combination of the different views. This limitation of single parameter radiomics of being unable to capture true textural information have been explored in a limited fashion. For example, recent reports have shown the extension of conventional single image radiomics and the Gray-level Co-occurrence Matrix (GLCM) into a joint intensity matrix (JIM) by capturing the joint textural information in two imaging parameters that are plotted simultaneously in joint distribution graph [22]. Chaddad et al. demonstrated that the JIM method outperformed conventional radiomic GLCM in Gleason histological score prediction (G1–G3) of prostate cancer from mpMRI consisting of *T*_2_-weighted images and ADC mapping. The JIM plus GCLM resulted in higher AUCs of Gleason score ranging from 0.78 (G1), 0.82 (G2), and 0.65 (G3). However, the JIM is an extension of radiomics to only two imaging parameters, and may not be generalizable to several multiparametric imaging parameters, thereby limiting the method to only two types of radiological input of images. Other imaging modalities have used probabilistic methods to combine different features for better characterization of tissue types, Mojabi et al. used ultrasound (US) and microwave (MW) to create probability maps based on Bayesian methods on the quantitative images from US and MW from known breast phantoms [27]. The phantoms provided information about the tissue properties and they defined these maps as the composite tissue-type-image (cTTI) for both single modality and combined modalities from US and MW methods. To construct the TTIs, they needed at least two properties from the object of interest (OI) and the probability distribution functions (PDF) for the OI. All the tissue properties from the cTTI allowed for better discrimination of each tissue type [27].

Therefore, texture analysis on the complete multiparametric datasets would provide information about the “true texture” of the tissue rather than from a single specific point of view. To that end, we developed a multiparametric radiomics imaging framework (mpRad) for integrating and analyzing the information present in multiparametric and multimodal radiological data [28]. In this paper, we introduce five new techniques for analyzing the texture of multiparametric imaging datasets and evaluate and validate these techniques on clinical breast mpMRI datasets.

## Materials and methods

### Theory

#### The radiomic tissue signature model

We define a tissue signature (TS) that represents the composite feature representation of a tissue type based each of the different imaging sequences and demonstrated in Fig. 2. Mathematically, for N different imaging parameters with TS at a voxel position, *p*, *S*_p_ is defined as a vector of gray-level intensity values at that voxel position, *p* across all the (*N*) images in the data sequence for different tissue types and is given by the following equation,

1 $$S_{{\text{p}}} \, = \,\left[ {I_{{\text{p}}}^{\left( 1 \right)} ,\,I_{{\text{p}}}^{\left( 2 \right)} ,\,I_{{\text{p}}}^{\left( 3 \right)} ,\, \ldots ,\,I_{{\text{p}}}^{\left( N \right)} } \right]^{T}$$

where, *I*_*p*_ is the intensity at voxel position, *p* on each image, and *T* corresponds to the transpose operation.

#### The tissue signature probability matrix features

The tissue signature probability matrix (TSPM) characterizes the spatial distribution of tissue signatures within a ROI. The mathematical formulation of TSPM is defined as: Suppose that the intensity values representing each voxel are quantized to some *G* level, then the total number of possible tissue signatures in a dataset consisting of *N* images will be equal to ${G}^{N}$. We define a function *f*:*T* → *M*, where *T* is the set of all tissue signatures in the dataset and *M* is a *N*-dimensional matrix with edges of length *G* where each tissue signature is represented as a cell. The function *f* populates each cell of the matrix *M* with the frequency of occurrence of the corresponding tissue signature in the set T. The resulting matrix *M* is called the tissue signature probability matrix (TSPM). The information content of the N-dimensional multiparametric imaging dataset $({X}_{1},{X}_{2},\dots {X}_{N})$ can be analyzed by computing the joint entropy, uniformity, and mutual information of the resultant TSPM [29]. These features are defined below.

1. The TSPM entropy, *H* is given by the following equation:
  2 $$H\left( {X_{1} ,X_{2} , \ldots ,X_{N} } \right) = - \mathop \sum \limits_{{i_{1} = 1}}^{G} \mathop \sum \limits_{{i_{2} = 1}}^{G} \ldots \mathop \sum \limits_{{i_{N} = 1}}^{G} {\text{TSPM}}\left( {i_{1} ,i_{2} , \ldots ,i_{N} } \right)\log_{2} {\text{TSPM}}\left( {i_{1} ,i_{2} , \ldots ,i_{N} } \right)$$
2. The TSPM uniformity, *U* is given by the following equation:
  3 $$U\left( {X_{1} ,X_{2} , \ldots ,X_{N} } \right) = \mathop \sum \limits_{{i_{1} = 1}}^{G} \mathop \sum \limits_{{i_{2} = 1}}^{G} \ldots \mathop \sum \limits_{{i_{N} = 1}}^{G} {\text{TSPM}}\left( {i_{1} ,i_{2} , \ldots ,i_{N} } \right)^{2}$$
  Here, $TSPM\left( {i_{1} ,i_{2} , \ldots ,i_{N} } \right)$ represents the value of the cell located in the position $\left( {i_{1} ,i_{2} , \ldots ,i_{N} } \right)$ of the N-dimensional TSPM matrix, and N corresponds to the number of different imaging parameters.
3. The TSPM mutual information, *MI* is given by
  4 $$MI\left( {X_{1} ;X_{2} ; \ldots ;X_{N} } \right) = \left( {H\left( {X_{1} } \right) + H\left( {X_{2} } \right) + \ldots + H\left( {X_{N} } \right)} \right) - \ldots + \ldots \left( { - 1} \right)^{N - 1} H\left( {X_{1} ,X_{2} , \ldots ,X_{N} } \right)$$

By choosing different possible subsets $Y \subseteq \left\{ {X_{1} ,X_{2} , \ldots ,X_{N} } \right\}$ and different values of H(Y), U(Y), and MI(Y) can be obtained producing a large number of mpRad features.

#### Tissue signature first order statistics features

The tissue signature first order statistics (TSFOS) features characterize the distribution of voxel intensities across all the imaging parameters. This is similar to a traditional first order histogram, except, the TSFOS histogram is computed from the voxel intensities across all the imaging sequences, which can be very useful when analyzing multiparametric imaging sequences, such as DWI, DCE, and PWI in certain applications. Let the tissue signature histogram (TSH) represent a TSFOS histogram that is computed by dividing the voxel intensities in mpMRI into *B* equally spaced bins. The first order statistical features (e.g., entropy) can be computed from the $TSH$ using the following equation:

5 $$Entropy_{TSFOS} = - \mathop \sum \limits_{i = 1}^{B} TSH\left( i \right)\log TSH\left( i \right)$$

where, (*i*) is for each image sequence.

The TSFOS histogram bins the intensities from all the imaging features together. As a result, the TSFOS histogram is very effective for decoding tissue characteristics in imaging sequences that encode an intrinsic inter-parametric relationship, for example, DWI, DCE, and PWI. The remaining TSFOS features, such as uniformity and energy, are derived in a similar method from the TSH.

#### Tissue signature co-occurrence matrix features

The tissue signature co-occurrence matrix (TSCM) characterizes the spatial relationship between tissue signatures within a ROI. The TSCM is defined similar to the gray-level co-occurrence matrix (GLCM) by using two input parameters, distance (d), and angle ($\theta$) between two tissue signature locations [14]. Mathematically, the GLCM between any two tissue signatures, S_i_ and S_j_ is given by the following equation

6 $$GLCM_{d}^{\theta } \left( {S_{i} ,S_{j} , m,n} \right) = \left| {\left\{ {r :S_{i} \left( r \right) = m, S_{j} \left( r \right) = n} \right\}} \right| \forall m,n \smallint \left\{ {1,2,3, \ldots ,G} \right\}$$

where $r \in N \left( {{\text{number}}\,{\text{of}}\,{\text{imaging}}\,{\text{sequences}}} \right)$ and |…| denotes the cardinality of a set.

Given a distance, *d* and angle, ($\theta$), the TSCM co-occurrence matrix for all such possible pairs of tissue signatures is given as follows:

7 $${\text{TSCM}}_{{\text{d}}}^{\theta } \left( { m,n} \right) = \Sigma_{i,j} {\text{GLCM}}_{{\text{d}}}^{\theta } \left( {S_{{\text{i}}} ,S_{{\text{j}}} , m,n} \right)\quad \forall i,j {\text{satisfied }}\,{\text{by}}\,{\text{d}}\,{\text{and}}\,\theta$$

Here, ${\text{TSCM}}_{{\text{d}}}^{\theta }$ is the tissue signature co-occurrence matrix. The TSCM can then be analyzed to extract twenty-two different TSCM features using the equations developed by Haralick et al. [13, 19].

#### Tissue signature complex interaction network analysis features

The tissue signature complex interaction network (TSCIN) characterizes the complex interactions that define the inter-parametric relationships between different imaging parameters based on statistical analysis. The TSCIN features are extracted by transforming a high-dimensional multiparametric radiological imaging data into a radiomic feature map using first or higher order statistical analysis of the tissue signature vectors, *S*_p_ at each voxel position. The TSCIN feature maps are then transformed into a single radiomic quantitative value corresponding to a ROI by using the summary statistical metrics of mean, median, or standard deviation.

##### First order TSCIN features

The first order TSCIN features are straightforward and calculated directly from the tissue signatures. For example, the TSCIN entropy at a voxel position, *p* is given by the following equation:

8 $${\text{Entropy}}_{{{\text{TSCIN}}}} \, = \,{\text{entropy}} \left( {S_{{\text{p}}} } \right)$$

The other first order TSCIN features are defined in a similar fashion from the first order histogram definitions.

##### Second order TSCIN features

The second order TSCIN features characterize the inter-parameter relationship within the tissue signature by computing a TSCIN relationship matrix (TSRM). Mathematically, the TSRM for a *N*-dimensional tissue signature at voxel position, *p* with *N* imaging sequences that are quantized to *G* gray levels is given by the following equation:

9 $${\text{TSRM}}_{{\text{d}}}^{{\text{p}}} \left( {i,j} \right) = \left| {\left\{ {k:I_{{\text{p}}}^{{\left( {\text{k}} \right)}} = i, I_{{\text{p}}}^{{\left( {\text{k } + \text{ d}} \right)}} = j} \right\}} \right| \forall i,j \in \left\{ {1,2,3, \ldots ,G} \right\}, k \in \left\{ {1,2, \ldots ,N} \right\}$$

Here, *d* represents the distance between the two imaging parameters, I^(k)^ and I^(k+d)^.

**Fig. 1:**
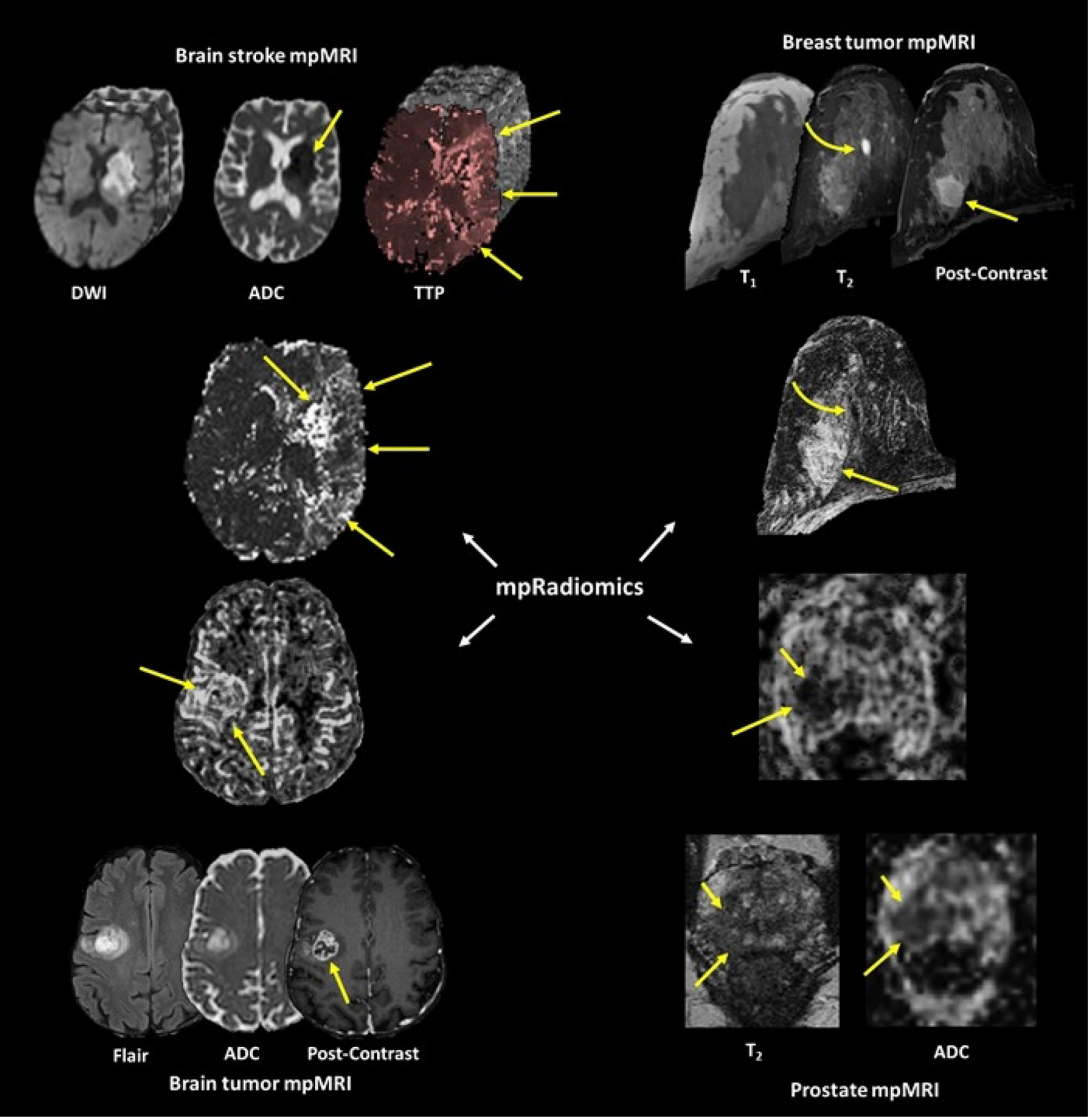
Illustration of the mpRad framework applied to different organs for analysis of different pathologies

**Fig. 2:**
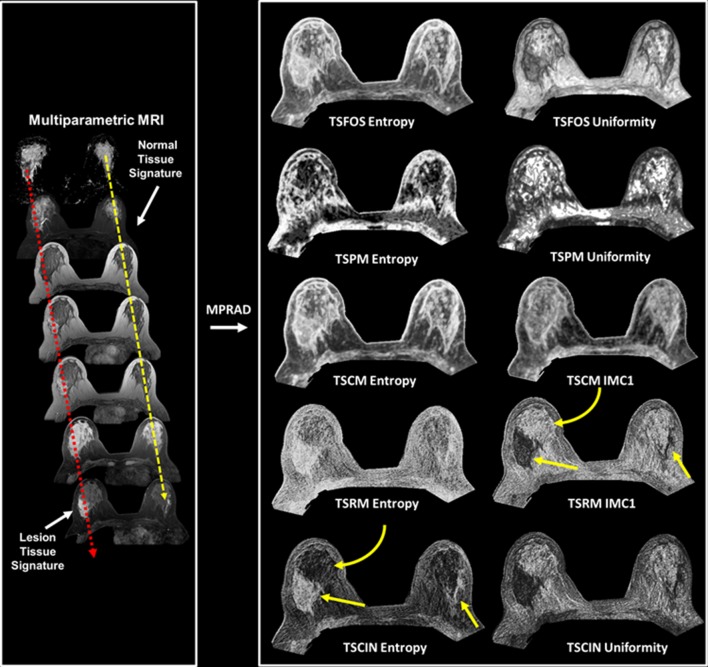
Illustration of the five different types of multiparametric radiomics (mpRad) framework features based on first and second order statistical analysis. Left: Construction of representative breast tissue signatures on normal and lesion tissue. Right**:** mpRad features defined as the radiomic tissue signature first order statistics (TSFOS), tissue signature probability matrix (TSPM), and the tissue signature co-occurrence matrix (TSCM) features evaluate the complex interactions between different tissue signatures. The tissue signature complex interaction network (TSCIN) first order statistics and tissue signature relationship matrix (TSRM) features evaluate the inter-parameter complex interactions. The straight yellow arrows indicate the lesion tissue and the curved yellow arrow show glandular tissue

The TSRM is dependent on the relative location of different imaging parameters within the tissue signature. Consequentially, TSRM requires the input imaging series to have an intrinsic relationship between the different imaging sequences, for example, pharmacokinetic dynamic contrast enhanced (PK-DCE) imaging and diffusion-weighted imaging (DWI) sequences. The structure of the TSRM is similar to a G x G gray-level co-occurrence matrix, thereby, allowing us to utilize all the twenty-two equations established to extract relevant features from such matrices [13]. Figure 1 demonstrates the mpRad framework on four different mpMRI applications, breast, prostate, stroke, and brain cancer. Five classes of the mpRad features developed in this manuscript are illustrated in Fig. 2 from a representative breast mpMRI dataset.

## Clinical data

### Clinical data

#### Informed consent

All procedures performed in studies involving human participants were in accordance with the ethical standards of the institutional and/or national research committee and with the 1964 Helsinki declaration and its later amendments or comparable ethical standards. The studies are in accordance to the institutional guidelines for clinical research under IRB approved protocol by our institution for this retrospective study and informed consent of the subjects was waived.

#### Clinical breast data set

One hundred and thirty-eight patients with breast lesions were scanned using mpMRI. Lesion characteristics, molecular phenotypes, and lesion size for the patients were obtained. MRI scans were performed on a 3 T magnet (Philips), using a dedicated phased array breast coil with the patient lying prone with the breast in a holder to reduce motion. Briefly, the mpMRI sequences were T_1_WI, T_2_WI, DWI, pharmacokinetics (PK) DCE (15 s temporal resolution), and post-contrast high-resolution images. MRI sequence parameters were: An ultrafast spoiled gradient echo (T_1_-TFE) T_1_-weighted images (TR/TE: 5.37/2.3 ms; Slice thickness (ST) = 3 mm; Field of view(FOV): 35 cm x 35 cm; Flip angle(FA) = 120), fat-suppressed(FS) T_2_-weighted spin echo images (TR/TE: 6122/70 ms; ST = 4 mm; FOV:35 cm x 35 cm; FA = 900). The DCE-MRI was obtained using FS and non-FS, 3D FSPGR T1-weighted (TR/TE = 4.2/2.1 ms; FOV:35cmx35cm; ST = 5 mm) sequences. One non-FS pre- and fourteen post-contrast images (15 secs per acquisition) for PK analysis were obtained after intravenous administration via a power injector at a rate of 2 mL/sec of a Gadopentetic acid (Gd-DTPA) contrast agent (0.2 mL/kg(0.1 mmol/kg)) [30, 31]. Two minutes of T_1_ fat-suppressed high temporal resolution (15 s per acquisition) imaging was obtained to capture the wash-in phase of contrast enhancement, followed by a high spatial resolution scan for two minutes. Diffusion-weighted imaging was obtained using an FS spin-echo Echo Planar Imaging (EPI) sequence (TR/TE = 5000/90 ms, SENSE = 2, ST = 3-4 mm, b = 0–600 s/mm^2^) on three planes. Apparent Diffusion Coefficient (ADC) of water maps were constructed from the DWI. For registration, the DCE post-contrast images were used as the reference volume. The registration methods used on the breast MRI have been detailed in [32].

#### Pharmacokinetic (PK) contrast enhancement parameters

PK-DCE provides metrics of the vascularity of differnet breast tissue types. The PK-DCE quantitative metrics derived were the volume transfer constant (K^trans^ (min^−1^)) and the fractional volume of the extracellular extravascular space (EVF (V_e_)) for this study.

### Multiparametric radiomic analysis

Radiomic image maps and features were computed by filtering the mpMRI images with statistical kernels based on the first order TSFOS, TSPM, and TSCIN (e.g. entropy) features and second order TSCM and TSRM features (Haralick’s gray-level co-occurrence matrix features) described above. The optimal neighborhood and gray-level quantization values for filtering were determined by the image resolution, bit depth of the radiological images, empirical analysis of the uniformity, and the noise within the radiomic maps. The radiomic parameters of neighborhood and gray-level quantization were set to 5 × 5 with 128 gray levels for the mpMRI breast datasets. The ROIs from the different tissue types were segmented and overlaid on the mpRad maps for quantification of the texture values. The same ROIs were overlaid onto the ADC maps and PK DCE parameters for quantitative metrics.

The mpRad processing code was written in MATLAB and optimized to maximize the computational running time of the code using parallelization methods. The efficacy of mpRad code was evaluated on different processing platforms in computing mpRad feature maps. The cost of transferring the data to and from the GPU processors were included in the calculations. The mpRad feature mapping of the different parameters were further evaluated for computational complexity across multiple computational platforms with and without GPUs: Tesla K40c (12 GB RAM), Quadro P6000 (24 GB RAM), and the Nvidia DGX machine with four Voltas (132 GB RAM), and a CPU: Intel® Xeon® CPU E5-2643 (3.50Ghz) with a 64 GB RAM.

### Classification

We used the IsoSVM [8] feature embedding and classification framework for classifying benign from malignant lesions. The IsoSVM algorithm comprises of two component algorithms, the Isomap, and SVM [33, 34]. Briefly, the Isomap algorithm is a non-linear dimension reduction algorithm based on the geodesic distance and multidimensional scaling. The SVM algorithm is a linear binary classification algorithm that attempts to create a hyperplane that best separates different groups. The application of Isomap algorithm prior to SVM transforms the high-dimensional mpRad feature space into a linearly separable space. Then, the SVM algorithm trains a classification model to classify between benign and malignant patients on the transformed feature space. The imbalance in the number of benign and malignant patients was resolved by setting a higher misclassification cost for benign than malignant patients when training the SVM classifier. We determined the optimal value of the misclassification penalty using a grid search on misclassification penalty ratios from the set:

10 $${\text{Benign}}:{\text{Malignant}} = \left\{ {1{:}1,{ }1.5{:}1,{ }2{:}1,{ }2.5{:}1,{ }3{:}1,{ }3.5{:}1,{ }4{:}1} \right\}.$$

In a grid search, each of the different misclassification penalty ratios in the above set are tested in a leave-one-out cross validation setting and the misclassification penalty that achieves the maximum AUC was chosen as the optimal misclassification penalty [35].

### Statistical analysis

Summary statistics (mean and standard error of the mean) were calculated for each quantitative MRI and mpRad feature. The sample size of the training data set was calculated based on the ROC curve [36, 37]. A sample size of 112 subjects can give 85% power to detect a specificity of at least 80% (under significance level alpha = 5%). The same sample size also gives us greater than 85% power to differentiate sensitivities between 80 and 95% at alpha = 5% significance level. An unpaired t-test (two-sided) was performed to compare the mpRad features for different breast tissue types of normal glandular tissue, benign, and malignant lesions. Univariate logistic regression analysis was used to find associations between the mpRad features and the final diagnosis. Sensitivity, specificity, and receiver operating characteristic (ROC) and Areas under the ROC curve (AUC) analysis were performed to assess diagnostic performance of the mpRad parameters. Statistical significance was set at *p*  < 0.05.

## Results

**Fig. 3:**
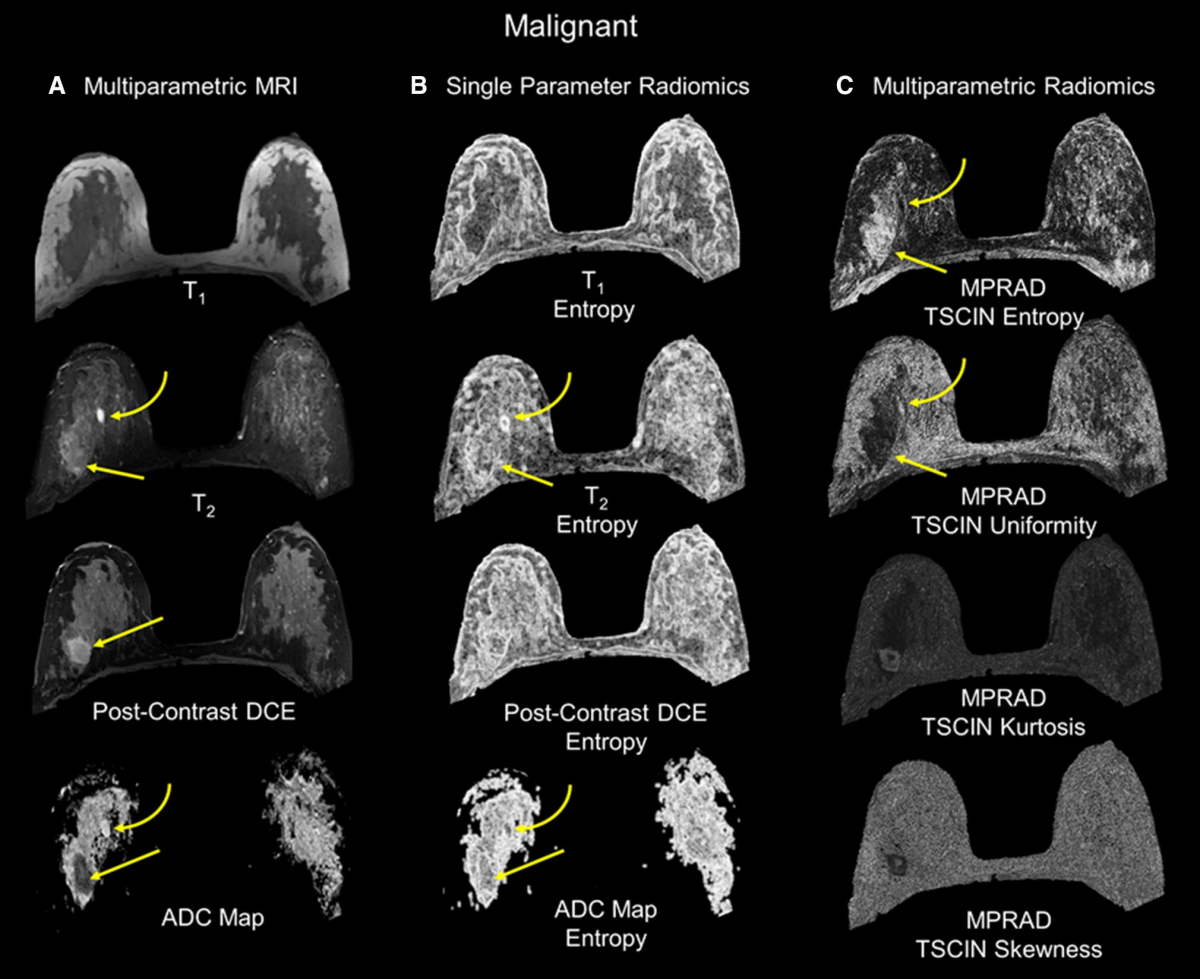
The radiomic feature maps (RFM) obtained from single and multiparametric radiomics (mpRad) analysis in a patient with a malignant lesion. The straight yellow arrow highlights the lesion location. The curved arrow demonstrates a benign cyst in the breast. **a** Multiparametric MRI parameters used for the mpRad framework. **b** Single radiomic gray-level co-occurrence matrix (GLCM) entropy features maps from each MRI parameter. **c** The mpRad RFMs tissue signature co-occurrence matrix (TSCM) and tissue signature complex interaction network (TSCIN) radiomic features. Note, the improved tissue delineation between the different tissue types using the mpRad framework

The mean age of the patients was 52 ± 11 years ranging between 24 and 80 years. Of the 138 patients, there were 97 patients with biopsy proven malignancy and 41 patients had benign lesions. Table 1 summarizes the lesion characteristics and molecular phenotypes for the patients. The average tumor size ranged from 2.3 to 3.2 cm in size. The ADC map and PK-DCE metrics were significantly different between benign and malignant lesions. Figure 3 illustrates both single and mpRad feature maps from a representative patient with a malignant lesion in the upper outer quadrant of the right breast with a benign appearing cyst superior and more medial to the lesion (curved yellow arrow). The cyst is uniformly bright on T2 and the ADC map consistent with known MRI tissue characteristics associated with cysts. Similarly, the cyst is dark on T1 with negative contrast enhancement on the DCE image indicating lack of vascularity. Moreover, the lesion tissue appears to heterogenous on the MRI images with a decreased ADC value and increased PK-DCE characteristics. The single radiomic images exhibit some texture features, however, compared to mpRad radiomic images, there is a striking difference in the textural representation of both normal and lesion tissue. In particular, the cyst has decreased entropy in the mpRad compared to single radiomic images. The lower entropy in the cyst is consistent with the fact, that the homogenous object has less disorder and hence lower entropy. This is clearly evident when looking at the lesion which is heterogenous and higher entropy values.

**Table 1:** Summary of demographic and clinical data

Malignant characteristics	IDC	IDC + DCIS	IDC + ILC	ILC	Others
	*N* = 29 (30%)	*N* = 34 (35%)	*N* = 17 (18%)	*N* = 12 (12%)	*N* = 5 (5%)
Age, years^a^	50 ± 12	55 ± 8	50 ± 11	55 ± 8	56 ± 9
Tumor size (cm)	3.2 ± 2.1	2.3 ± 1.7	2.8 ± 1.3	2.9 ± 2.0	2.9 ± 1.7
Phenotypes					
Luminal A^b^	18	15	8	11	2
Luminal B^b^	1	9	7	1	2
HER2+ ^b^	4	2	0	0	0
Triple negative^b^	6	8	2	0	1

Benign characteristics	Benign breast tissue	Stable imaging	Fibroadenoma	Sclerosing adenosis	Papilloma	Fibrocystic changes
	*N* = 14 (35%)	*N* = 10 (24%)	*N* = 10 (24%)	*N* = 3 (7%)	*N* = 2 (5%)	*N* = 2 (5%)
Age, years^a^	50 ± 7	53 ± 13	46 ± 14	47 ± 13	46 ± 8	48 ± 2

*DCIS* ductal carcinoma in situ, *ILC* invasive lobular carcinoma, *LCIS* lobular carcinoma in situ, *IDC* invasive ductal carcinoma, *HER2* + human epidermal growth factor receptor 2

^a^Data are presented as mean ± (standard deviation)

^b^Data are presented as number of cases

**Fig. 4:**
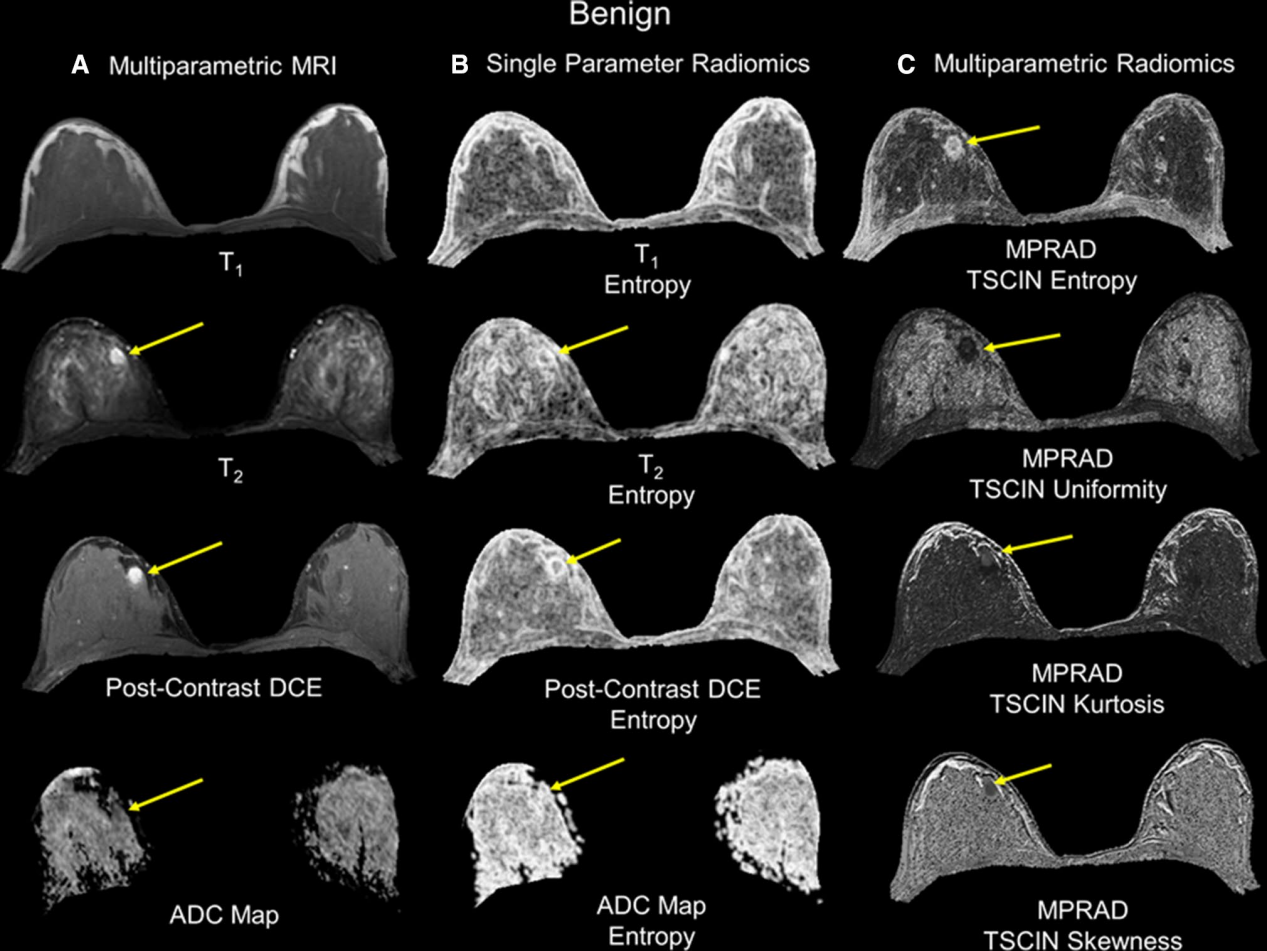
The radiomic feature maps (RFM) obtained from single and multiparametric radiomics (mpRad) analysis in a patient with a benign lesion. The straight yellow arrow highlights the lesion location. **a** Multiparametric MRI parameters used for the mpRad framework. **b** Single radiomic gray-level co-occurrence matrix (GLCM) entropy features maps from each MRI parameter. **c** The mpRad RFMs tissue signature co-occurrence matrix (TSCM) and tissue signature complex interaction network (TSCIN) radiomic features

Figure 4 illustrates both the single and mpRad feature maps from a representative benign patient. Again, there was a clear difference between the textural representation of the lesion and glandular tissue using mpRad. Furthermore, the tissue characterization of lesion and glandular tissue was consistent for both the benign and the malignant patients. Table 2 summarizes the quantitative values from each single parameter, TSPM entropy, and AUCs for the individual and mpRad features on benign and malignant lesions demonstrating improved tissue characterization using mpRad. The mpRad TSPM entropy was computed using all the MRI parameters and was significantly different between benign and malignant lesions (Benign:7.06 ± 0.27, Malignant:8.93 ± 0.17, *p* < 0.00001). Furthermore, the AUC of TSPM entropy was 0.82, 9% higher than the maximum AUC (0.75 for post-contrast DCE) obtained from univariate analysis of first order entropy computed from different imaging parameters. More importantly, there were no significant differences between the contralateral glandular tissue in benign and malignant cases for both the single and multiparametric radiomic features as shown in Table 3.

**Table 2:** Single and multiparametric entropy values corresponding to benign and malignant breast tumors

	Benign tumor	Malignant tumor	*p* value	AUC
MRI metrics				
ADC map values (× 10^–3^ mm^2^/s)	1.89 ± 0.10	1.15 ± 0.03	0.0001	
*K*^trans^ (1/sec)	0.27 ± 0.05	0.80 ± 0.32	0.005	
Single parameter entropy				
Entropy T1	4.14 ± 0.11	4.66 ± 0.06	0.00008	0.72 (0.64–0.79)
Entropy T2	4.98 ± 0.12	5.42 ± 0.06	0.002	0.68 (0.59–0.75)
Entropy b0	4.44 ± 0.17	5.06 ± 0.09	0.002	0.67 (0.59–0.75)
Entropy b600	3.00 ± 0.20	3.77 ± 0.09	0.0009	0.67 (0.59–0.75)
Entropy ADC	4.90 ± 0.12	5.40 ± 0.06	0.0004	0.70 (0.62–0.77)
Entropy post-contrast DCE (High spatial resolution)	5.00 ± 0.10	5.54 ± 0.05	0.00001	0.75 (0.67–0.82)
Entropy PK-DCE Pre	4.32 ± 0.12	4.65 ± 0.05	0.02	0.62 (0.54–0.70)
Entropy PK-DCE post (wash-in)	4.89 ± 0.08	5.30 ± 0.05	0.00006	0.72 (0.64–0.79)
Entropy PK-DCE post (wash-out)	4.90 ± 0.09	5.24 ± 0.04	0.00007	0.69 (0.60–0.76)
Multiparametric entropy				
TSPM entropy (all Parameters)	7.06 ± 0.27	8.93 ± 0.17	< 0.00001	0.82 (0.74–0.88)
TSPM entropy (PK-DCE)	7.06 ± 0.27	8.92 ± 0.17	< 0.00001	0.82 (0.74–0.88)
TSPM entropy (high spatial resolution DCE)	6.74 ± 0.19	8.28 ± 0.12	< 0.00001	0.82 (0.75–0.88)
TSPM entropy (DWI)	6.66 ± 0.22	8.20 ± 0.15	< 0.00001	0.78 (0.70–0.85)

*DWI* diffusion-weighted imaging, *ADC* apparent diffusion coefficient, *PK* pharmacokinetic, *DCE* dynamic contrast enhancement, *FOS* first order statistics, *TSPM* tissue signature probability matrix

**Table 3:** Single and multiparametric entropy contralateral glandular tissue values from patients with benign and malignant breast tumors

	Glandular tissue benign patients	Glandular tissue malignant patients	*p* value
Single parameter entropy			
Entropy T1	5.29 ± 0.11	5.12 ± 0.06	0.20
Entropy T2	5.37 ± 0.10	5.32 ± 0.06	0.68
Entropy b0	5.19 ± 0.24	4.89 ± 0.10	0.27
Entropy b600	3.46 ± 0.24	3.13 ± 0.10	0.20
Entropy ADC	5.27 ± 0.28	5.39 ± 0.16	0.71
Entropy post-contrast DCE (high spatial resolution)	5.13 ± 0.10	5.00 ± 0.06	0.26
Entropy PK-DCE pre	5.24 ± 0.12	5.12 ± 0.05	0.38
Entropy PK-DCE post (wash-in)	5.28 ± 0.11	5.18 ± 0.05	0.40
Entropy PK-DCE post (wash-out)	5.30 ± 0.10	5.24 ± 0.05	0.60
Multiparametric entropy			
TSPM entropy (all Parameters)	10.93 ± 0.34	10.64 ± 0.17	0.46
TSPM entropy (PK-DCE)	10.92 ± 0.34	10.64 ± 0.17	0.47
TSPM entropy (high spatial resolution DCE)	9.17 ± 0.17	9.04 ± 0.10	0.51
TSPM Entropy (DWI)	9.31 ± 0.35	9.06 ± 0.18	0.54

*DWI* diffusion-weighted imaging, *ADC* apparent diffusion coefficient, *PK* pharmacokinetic, *DCE* dynamic contrast enhancement, *FOS* first order statistics, *TSPM* tissue signature probability matrix

**Fig. 5:**
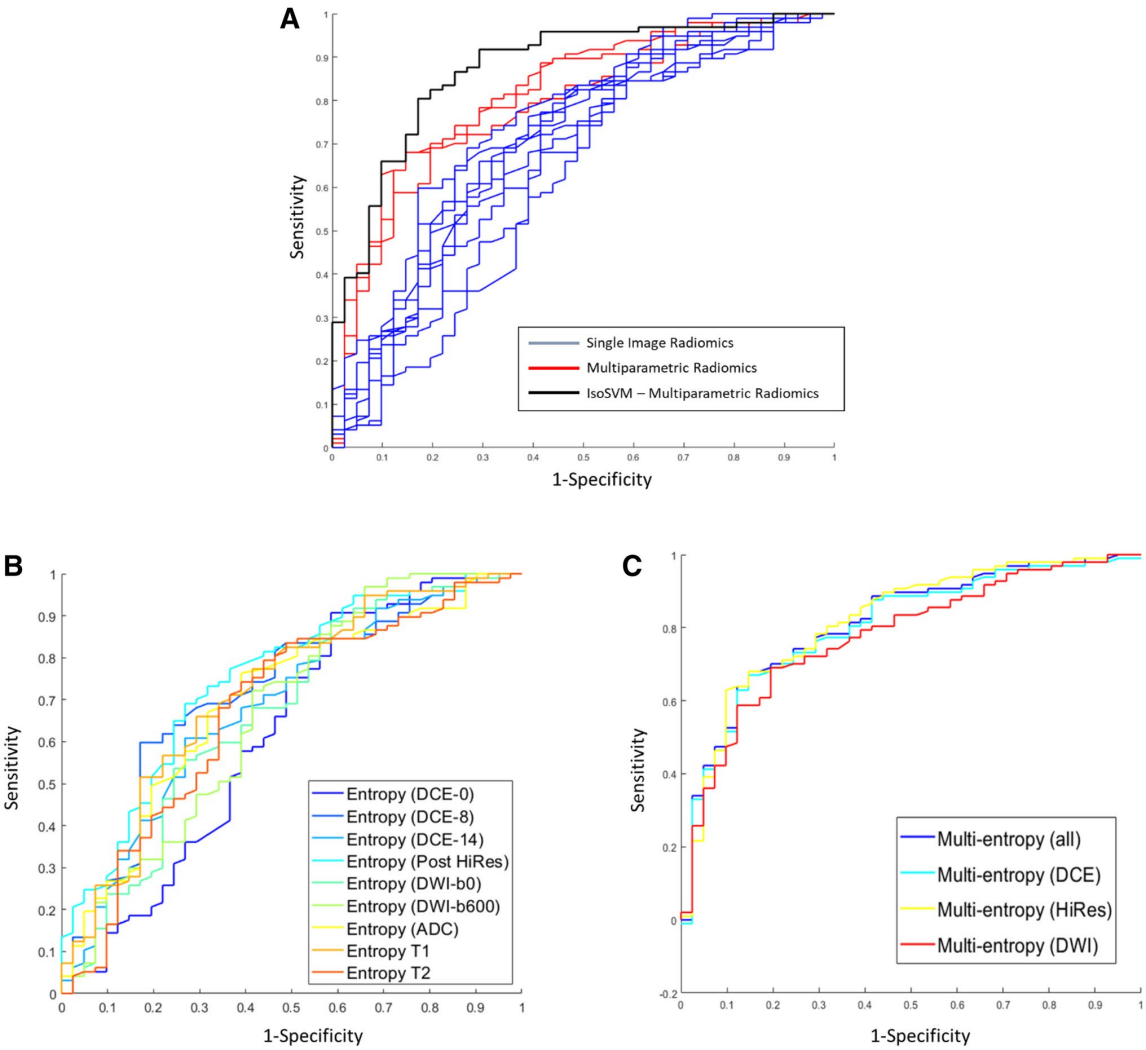
The predictive accuracy between the single parameter based radiomics features and multiparametric radiomics (mpRad) features using receiver operating characteristic (ROC) curve analysis is demonstrated. **a** The AUC for IsoSVM was 0.87 and shown on the left and displayed in black. The mpRad feature ROC curves (displayed in red) produced area under the ROC curve (AUC) values that were 9–28% greater than the AUCs obtained for single parameter radiomics (ROC curves displayed in blue). **b** The AUC curves for the each mpRAD feature are shown in the middle. The AUC values for these features ranged from 0.78 to 0.82. **c** The single radiomic AUC curves for each feature are shown on the right and ranged from 0.62 to 0.75

The top mpRad features for differentiating benign from malignant lesions are summarized in Table 4. Using the IsoSVM with leave-one-out cross validation with the top mpRad features produced a sensitivity and specificity of 82.5% and 80.5%, respectively, with an AUC of 0.87 (0.81–0.93). The optimal IsoSVM parameters were k = 20, d = 1 with an imbalance ratio of 3:1 of benign to malignant. The predictive power of the single radiomic, mpRad radiomic, and the IsoSVM models are shown in Fig. 5. The resulting ROC curves demonstrated superior discrimination between benign and malignant lesions from the mpRad radiomic methods compared to single radiomics as shown in Fig. 5.

**Table 4:** Top multiparametric radiomic features for classification of malignant from benign breast tumors

S. No	mpRad radiomic feature	Benign tumor	Malignant tumor	*p* value	AUC
1	TSPM entropy (all parameters)	7.06 ± 0.27	8.93 ± 0.17	< 0.00001	0.82 (0.74–0.88)
2	TSPM entropy (DCE)	7.06 ± 0.27	8.92 ± 0.17	< 0.00001	0.82 (0.74–0.88)
3	TSPM entropy (HiRes)	6.74 ± 0.19	8.28 ± 0.12	< 0.00001	0.82 (0.75–0.88)
4	TSPM entropy (DWI)	6.66 ± 0.22	8.20 ± 0.15	< 0.00001	0.78 (0.70–0.85)
5	TSCIN DWI maximum	0.44 ± 0.02	0.34 ± 0.01	< 0.00001	0.77 (0.69–0.83)
6	TSCIN DWI standard deviation	0.18 ± 0.01	0.12 ± 0.00	< 0.00001	0.79 (0.71–0.85)
7	TSCIN DWI range	0.34 ± 0.02	0.24 ± 0.01	< 0.00001	0.79 (0.71–0.85)
8	TSCIN DWI median absolute deviation	0.13 ± 0.01	0.09 ± 0.00	< 0.00001	0.78 (0.70–0.84)
9	TSCIN DCE kurtosis	2.63 ± 0.14	3.37 ± 0.08	0.00004	0.76 (0.68–0.83)
10	TSCIN DCE skewness	− 0.69 ± 0.07	− 1.06 ± 0.04	0.00001	0.75 (0.67–0.82)

In the supplementary data, we demonstrate the application of mpRad to well characterized digital phantoms that are considered to be the gold standard for testing texture software [15, 38, 39]. These phantoms consisted of photographs of different objects with varying textural characteristics, ranging from rough to smooth and combinations of both. We were able to successfully distinguish the different texture features from the various objects in the Bordatz library with excellent accuracy between the two different phantoms.

For the computational complexity, as expected, the Nvidia DGX outperformed the other GPU and CPU processors with a time efficiency of approximately 12 min per complete mpMRI dataset per patient. The two Tesla K40Cs linked in parallel had a time efficiency of approximately 25 min per patient. Whereas, the Quadro P6000 (24 GB) had a time efficiency of approximately 35 min per patient. However, optimized software code for the high-end CPU took approximately 20 min per patient.

## Discussion

We have developed and validated a new multiparametric imaging radiomics (mpRad) framework that integrates all the radiological data to define different tissue texture characteristics. The mpRad features outperformed all the single radiomic features in the mpMRI breast dataset. The mpRad features captured the underlying tissue texture based on tissue signatures of each image, rather than individual imaging parameter intensities. More importantly, the mpRad method produces full texture images for visualization of normal and lesion heterogeneity, thereby providing radiologists with a new tool for visualization and quantization of the true underlying tissue heterogeneity in conjunction with traditional breast images.

In multiparametric imaging settings, single radiomic features from each individual image can result in very large numbers of texture features creating a high-dimensional dataset across all images for analysis. These single radiomic features may not reflect the true underlying tissue composition, heterogeneity, or homogeneity and only provide limited information corresponding to the physical modeling of that single imaging parameter.

The mpRad framework extracts radiomic features that considers the complete multiparametric dataset, hence producing more meaningful features and textural visualization of the underlying tissue overcoming the limitations of single parameter radiomics. MpRad provides a solution for potential sensitive and specific biomarkers of normal and tumor tissue for diagnostic and monitoring of patients.

Using the mpRad framework allows investigators to analyze the complex interactions between different imaging parameters and opening up a completely new source of information that did not exist with conventional radiomic features.

In the breast lesions, consistent with other reports in breast and other cancers, malignant breast lesions had increased entropy or heterogeneity compared to benign lesions [8, 22, 40, 41]. Importantly, no differences in the normal glandular tissue were noted between patients with either benign and malignant lesions [8, 22, 40, 41]. The mpRad radiomic feature maps delineated different tissue types better quantitively and qualitatively than any single radiomic feature map, for example, in cysts, normal, and peri-tumoral regions. Finally, the mpRad demonstrated excellent sensitivity and specificity with increased AUC metrics compared to single radiomic features. The IsoSVM mpRad AUCs were comparable with those AUCs discussed in the literature for discriminating benign from malignant lesions from radiologist [42, 43].

In general, multiparametric imaging for applications such as brain, breast, and prostate MRI produce large number of images corresponding to each slice location resulting in high-dimensional image space. Extracting radiomic features from each image individually in these types of datasets are time consuming and may not provide complete information about the lesion tissue. The mpRad framework resolves this potential issue by extracting radiomic features that not only analyze the progression of tissue texture with time but also evaluate the overall tissue texture over large data sets.

There are, however, some technical limitations to the use the mpRad in practice. First, there is a need for high-end graphical processor units (GPU) or CPUs with large memory and optimized software for processing which can be challenging to obtain or are very expensive to implement. The repeatability and reproducibility of radiomics have been demonstrated in both phantoms and clinical studies, the extension to mpRad would be straightforward and is currently under investigation [24, 44–47]. Our initial testing of the mpRad method using the Brodatz features are very encouraging and consistent with known radiomic features. More specific to the present study, any assessment of the clinical value of mpRad network will require prospective studies for validation of the mpRad methods. Although, a sample size was calculated for meaningful statistics, larger datasets, and prospective studies will be the real test for this method. These types of studies would have subsequent follow-up and pathological correlation for evaluation of the mpRad features in breast cancer patients. Ongoing studies using mpRad for radiomic characterization in both external and internal datasets are under investigation for determining treatment response from multisite locally advanced cancer clinical trials [48, 49]. This preliminary methods study was focused on development and characterization of the mpRad method on a large breast dataset with new radiomic features and identifying improved radiomic biomarkers linked to known biological MRI parameters of PK-DCE and ADC mapping of breast lesions.

In conclusion, we have demonstrated that mpRad framework shows excellent potential in analysis of textural information using multiparametric breast imaging data. These methods can be extended and used in different clinical applications beyond those presented in this work [50]. With increasing use of multiparametric imaging in clinical setting, mpRad provides an ideal framework for future clinical decision support systems.

## Code availability

Our software will be freely available to academic users after issue of pending patents and a materials research agreement is obtained from the university. Due to University regulations, any patent pending software is not available until a patent is issued.

## Electronic supplementary material

Below is the link to the electronic supplementary material.

Supplementary material 1 (DOCX 339 KB)

## Data Availability

All relevant clinical data are available upon request with adherence to HIPPA laws and the institutions IRB policies**.**
