# Supplementary material for: Multiparametric radiomics methods for breast cancer tissue characterization using radiological imaging
Source: Breast Cancer Res Treat. 2020 Feb 4;180(2):407–21. doi: 10.1007/s10549-020-05533-5 (PMC7066290; doi:10.1007/s10549-020-05533-5)
Supplement: Supplementary file 1 — Supplementary material 1 (DOCX 339 KB) [file 10549_2020_5533_MOESM1_ESM.docx]

**Supplementary material**

**Digital Phantom Data**

The multiparametric imaging radiomic feature extraction methods developed in this manuscript were tested on the well-known texture phantom from University Southern California (USC) shown in **Figure S1A and S1B** (1, 2). The texture phantom images consist of a composite mixture of several raw texture images of grass, sand, wool, water, and others derived by Brodatz (3). Using these composite images, ground truth texture images (**Figure S1-1A and S1A**) can be determined to demonstrate the effectiveness of any radiomic method (1, 2). The ground truth images are shown in the top row in **Figure S1**. We stacked a series of these texture phantom images (**figure S1-B1 and S1B2**) to demonstrate the effectiveness of the multiparametric radiomics (mpRad) tissue signature model to accurately segment each of the different textures. We applied single image radiomics to each image and mpRad to stacked images to compare the results from the two methods.

| 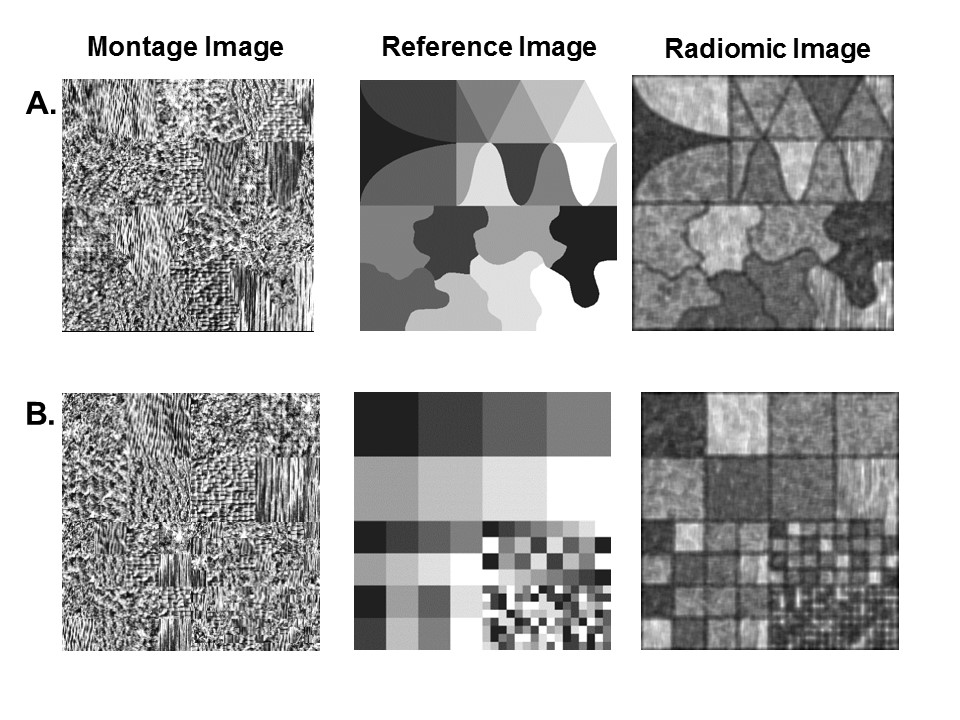 |
| --- |
| Figure S1. Example of the phantom images used for validation of the mpRadiomics methods. **A.** Montage image with different shapes with the reference and calculated radiomic image to the right. **B.** Montage image with different size rectangles and the calculated radiomic image to the right. |

**Results**

The mpRad features results on each USC composite texture image are shown in **Figure S1**. The reference texture ground truth images provide a method to evaluate the radiomic features from known objects with intrinsic high texture. From the two composite images, both single and multiparametric radiomic features were able to produce a 100 percent match with the reference images confirming the method on an independent data set (Figure S1A-B Column 3). **Figure S2** demonstrates that the mpRad was able to capture the differences in both shape and intensity distribution of both the single parameter radiomic images with excellent detail of the underlying structure. **Table S1 (a)** summarizes the single image entropy, uniformity and gray level co-occurrence matrix (GLCM) mutual information values for each of the texture classes in the two mosaics. As shown in **Table S1**, the each of the radiomic metric clearly separates the eight groups based on their underlying texture. **Table S2 (a-c)** summarizes the MPRAD TSFOS entropy, TSFOS uniformity and TSCM IMC1 values for each of the different texture class groups in the combined multiparametric mosaic shown in **Figure S2B.** The mpRad features demonstrate how the multidimensional texture of each of the different texture types when combined with another texture type with a different textural characteristic. For example, the entropy of the grass texture decreases from 7.07 to 6.73 when combined with water, which has a lower entropy of 6.16 in the multidimensional feature space. Moreover, the trend followed by the radiomic values extracted from different texture types stays consistent in the multidimensional radiomic space as demonstrated by the diagonal values of **Tables S2 (a-c).**

| 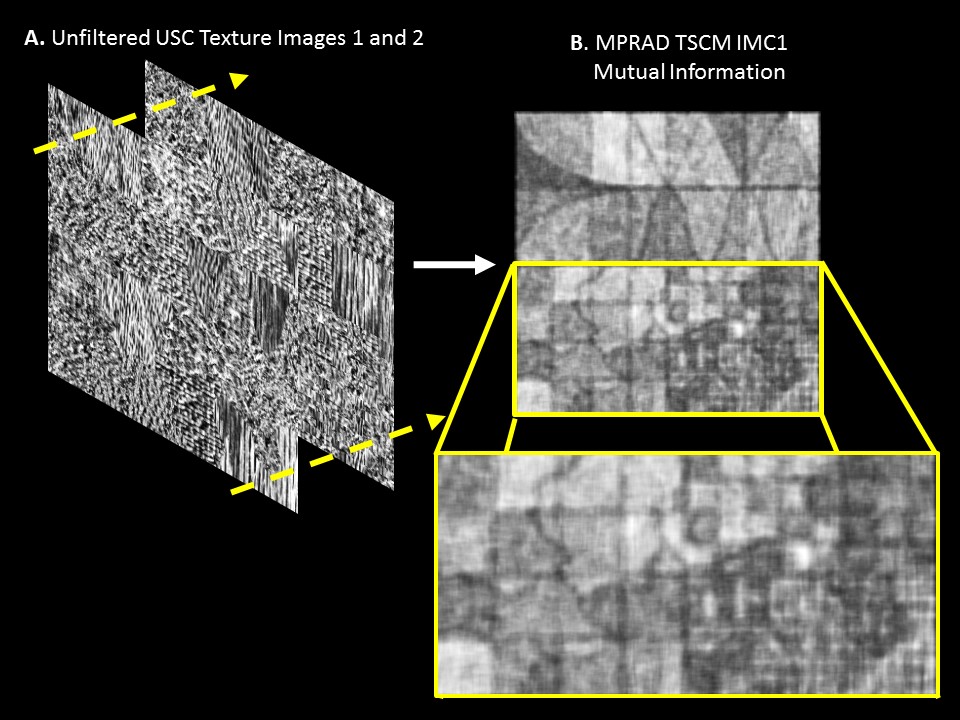 |
| --- |
| Figure S2. **A.** Multiparametric USC composite images with tissue signature vectors(yellow arrows) . **B.** The top image demonstrates the mpRadiomics (mpRad) image of the combined USC images. The bottom row shows an enlarged radiomic image from reference image. The mpRad was able to capture the differences in both shape and intensity distribution of the combined data from the single parameter radiomic images with excellent detail of the underlying structure. |

**References:**

1. Laws KI (1980) Rapid texture identification. *International Society for Optics and Photonics*:376-381.

2. Laws KI (1980) Textured Image Segmentation. USCIPI Report 940. PhD (University of Southern California).

3. Brodatz P (1966) *Textures: A Photographic Album for Artists and Designers.* (Dover Publications ).

**Table S1**. Summary of single image radiomic values (first order entropy, uniformity and gray level co-occurrence matrix mutual information) for each of the eight different texture types.

|  | Texture | Mosaic 1 | | | Mosaic 2 | | |
| --- | --- | --- | --- | --- | --- | --- | --- |
|  |  | Entropy | Uniformity | Mutual Information | Entropy | Uniformity | Mutual Information |
| 1 | Grass | 6.56 | 0.02 | -0.45 | 6.62 | 0.02 | -0.45 |
| 2 | Water | 5.38 | 0.03 | -0.27 | 5.40 | 0.03 | -0.25 |
| 3 | Sand | 6.13 | 0.02 | -0.37 | 6.02 | 0.02 | -0.35 |
| 4 | Wool | 6.04 | 0.02 | -0.35 | 6.01 | 0.02 | -0.33 |
| 5 | Pigskin | 6.05 | 0.02 | -0.35 | 6.02 | 0.02 | -0.33 |
| 6 | Leather | 6.51 | 0.01 | -0.42 | 6.31 | 0.03 | -0.42 |
| 7 | Raffia | 6.27 | 0.02 | -0.39 | 6.21 | 0.02 | -0.39 |
| 8 | Wood | 5.66 | 0.03 | -0.32 | 5.45 | 0.04 | -0.31 |

**Table S2**. Summary of multiparametric radiomic values for each of the different combination of texture types obtained by stacking the two images together.

**(a)** Tissue Signature First Order Statistics Entropy

| Entropy | Grass | Water | Sand | Wool | Pigskin | Leather | Raffia | Wood |
| --- | --- | --- | --- | --- | --- | --- | --- | --- |
| Grass | 7.07 | 6.73 | 7.01 | 6.98 | 6.96 | 7.26 | 7.12 | 6.70 |
| Water |  | 6.16 | 6.37 | 6.42 | 6.62 | 6.85 | 6.59 | 6.37 |
| Sand |  |  | 6.90 | 6.71 | 6.76 | 6.93 | 6.85 | 6.15 |
| Wool |  |  |  | - | 6.73 | 6.87 | 6.87 | 6.44 |
| Pigskin |  |  |  |  | 6.61 | 6.98 | 6.92 | 6.54 |
| Leather |  |  |  |  |  | 6.87 | 7.03 | 6.54 |
| Raffia |  |  |  |  |  |  | 6.95 | 6.74 |
| Wood |  |  |  |  |  |  |  | 6.49 |

**(b)** Tissue Signature First Order Statistics Uniformity

| Uniformity | Grass | Water | Sand | Wool | Pigskin | Leather | Raffia | Wood |
| --- | --- | --- | --- | --- | --- | --- | --- | --- |
| Grass | 0.02 | 0.02 | 0.02 | 0.01 | 0.02 | 0.01 | 0.01 | 0.02 |
| Water |  | 0.02 | 0.02 | 0.02 | 0.01 | 0.01 | 0.01 | 0.02 |
| Sand |  |  | 0.01 | 0.02 | 0.01 | 0.01 | 0.02 | 0.05 |
| Wool |  |  |  | - | 0.02 | 0.02 | 0.01 | 0.03 |
| Pigskin |  |  |  |  | 0.01 | 0.01 | 0.01 | 0.02 |
| Leather |  |  |  |  |  | 0.03 | 0.01 | 0.03 |
| Raffia |  |  |  |  |  |  | 0.01 | 0.02 |
| Wood |  |  |  |  |  |  |  | 0.02 |

**(c)** Tissue Signature Co-occurrence Matrix Mutual Information

| Mutual Information | Grass | Water | Sand | Wool | Pigskin | Leather | Raffia | Wood |
| --- | --- | --- | --- | --- | --- | --- | --- | --- |
| Grass | -0.44 | -0.39 | -0.42 | -0.41 | -0.41 | -0.44 | -0.43 | -0.42 |
| Water |  | -0.33 | -0.35 | -0.35 | -0.37 | -0.39 | -0.38 | -0.37 |
| Sand |  |  | -0.39 | -0.38 | -0.37 | -0.41 | -0.41 | -0.41 |
| Wool |  |  |  | - | -0.37 | -0.40 | -0.39 | -0.38 |
| Pigskin |  |  |  |  | -0.34 | -0.40 | -0.40 | -0.37 |
| Leather |  |  |  |  |  | -0.44 | -0.42 | -0.39 |
| Raffia |  |  |  |  |  |  | -0.41 | -0.40 |
| Wood |  |  |  |  |  |  |  | -0.38 |
